# The Effect of Wool Mulch on Plant Development in the Context of the Physical and Biological Conditions in Soil

**DOI:** 10.3390/plants12030684

**Published:** 2023-02-03

**Authors:** Katalin Juhos, Enikő Papdi, Flórián Kovács, Vasileios P. Vasileiadis, Andrea Veres

**Affiliations:** 1 Department of Agro-Environmental Studies, Hungarian University of Agriculture and Life Sciences, Villányi str. 29-43, H-1118 Budapest, Hungary; 2 Syngenta Crop Protection, 4058 Basel, Switzerland; 3 Agrologica Ltd., Puskin 15. II.10/B, H-1088 Budapest, Hungary

**Keywords:** β-glucosidase, DHA enzyme activity, labile carbon, straw mulch, plastic mulch, evapotranspiration coefficient, soil temperature

## Abstract

Mulching techniques can comprise a solution that better utilizes precipitation and irrigation water in such a manner that mitigates soil degradation and drought damage; however, there are still gaps in the literature with regard to the effect of the use of mulch materials on the development of plant–soil–microbe interactions. Waste fibers, as alternative biodegradable mulch materials, are becoming increasingly prominent. The effect of wool mulch (WM) on water use efficiency, with regard to pepper seedlings, was investigated in different soil types (sand, clay loam, peat) in a pot experiment. Two semi-field experiments were also set up to investigate the effect of WM–plant interactions on sweet pepper yields, as compared with agro textiles and straw mulches. Soil parameters (moisture, temperature, DHA, β-glucosidase enzymes, permanganate-oxidizable carbon) were measured during the growing season. The effect of WM on yield and biomass was more significant with the less frequent irrigation and the greater water-holding capacity of soils. Microbiological activity was significantly higher in the presence of plants, and because of the water retention of WM, the metabolic products of roots and the more balanced soil temperature were caused by plants. In the sandy soil, the straw mulch had a significantly better effect on microbiological parameters and yields than the agro textiles and WM. In soils with a higher water capacity, WM is a sustainable practice for improving the biological parameters and water use efficiency of soil. The effect of WM on yields cannot solely be explained by the water retention of the mulch; indeed, the development of biological activity and plant–soil–microbe interactions in the soil are also contributing factors.

## 1. Introduction

The pollution of natural resources, the rapid increase in food demand, and global warming are putting great pressure on the world’s water resources [1]. In some regions, drought causes significant economic, social, and environmental damage that seriously threatens food security [2,3]. Evaporative loss from land accounts for 20–40% of evapotranspiration globally, and 65% of these losses originate in the soil [4]. According to some estimates, 50–70% of annual precipitation returns to the atmosphere without being used for biomass production [5,6]; therefore, the lack of precipitation and high evaporation loss are contributing to the damage caused by global warming, and thus, this evapotranspiration loss must somehow be reduced [7]. From an economic agricultural production perspective, the question of how the amount of incoming precipitation and irrigation water can be preserved in the soil is important. This is a particularly critical issue for sandy soils with low water-holding capacities and poor structures; indeed, increasingly frequent droughts render it impossible to grow crops without irrigation. Wind erosion caused by a lack of water and weak soil structures causes further direct (sandblasting) and indirect damage (loss of nutrients and organic matter) to agriculture [8,9].

To a certain extent, surface cover techniques (mulching) can comprise a solution that better utilizes water and mitigates soil degradation damage. Various natural and artificial ground cover materials are used in agriculture and horticulture [1]. The type of mulch material greatly influences water retention and water utilization in soil during crop production [5]. Mulching with impermeable materials (e.g., plastic film) minimizes losses caused by evaporation; however, incoming precipitation cannot be utilized in the root zone. On the other hand, with porous mulch materials, precipitation reaches the roots; however, evaporation losses are greater when this material is used as compared with when the previously mentioned materials are used. Several studies have examined the soil moisture retention effect of different mulch materials and the efficiency of water use efficiency with regard to plants [10,11,12,13,14,15,16]. Their ability to suppress weeds [10,12,13,14,17,18] and the effects of mitigating soil erosion has also been investigated [10,19,20]; however, there is a need to specify these effects in accordance with soil parameters and the intensity level of the cropping system.

Instead of using synthetic mulch materials, alternative, natural, biodegradable mulch materials, which are often waste fibers from the textile industry (e.g., jute, wool mulch, various waste products from the cotton industry), are new potential tools that may be used in crop production [14,21,22,23]. In the textile industry, the demand for sheep wool has recently decreased, and sheared wool is often wasted [24]. Wool is rich in nutrients, especially nitrogen, and it has a good ability to retain moisture; this has proven to be an excellent mulch material in several experiments [15,17,18,25].

Cultivated plants are not the only organisms to need water in the soil; indeed, the water content of the soil also significantly affects microbial activity in the soil [26,27], and thus, mulching can be considered an important agrotechnical method for maintaining and improving soil health [22]. The use of appropriate mulch materials results in a more balanced microclimate, as well as better heat and water management; this significantly increases the number of soil microbes and their activity also increases [13,14,28,29,30,31,32,33]. The effectiveness of different mulch materials therefore significantly depends on the climatic and physical conditions in the soil, the irrigation methods used, and the water demand of the plants. For this reason, further research is needed to optimize mulching technologies that are applicable to local environmental conditions. According to our hypothesis, the effect of the use of mulch materials on crop yield can likely be explained by the efficiency of water utilization, in addition to the development of plant–soil–microbe interactions.

Moreover, little data is available on the biological effects of soil on alternative biodegradable mulch materials. Previous studies have focused primarily on evapotranspiration, soil temperature, and the effects of mulches on water utilization and yield. Hence, our objectives were as follows: (i) to examine the effect of wool mulch on plant development via changes in the physical parameters and biological parameters of different soil types, which were subjected to different levels of irrigation; and (ii) to examine the effect of wool mulch on the biological activity of soil and crop yields as compared with other mulch materials. When choosing the location for the experiments, we focused on the Carpathian and Mediterranean regions that are most affected by climate change.

## 2. Results

### 2.1. The Effect of Wool Mulch on Water Use Efficiency in Different Soil Types

We investigated the effect of wool mulch on evapotranspiration, biomass growth, and ET_c_ in different soil types in a short-term pot experiment. The results showed that the effect of the wool mulch treatment was different for each soil type (Table 1). Due to the short-term nature of the experiment, the differences in soil moisture between the two treatments (control and wool mulch) were not significant (loam soil: Wilks’ lambda = 0.16; *p* = 0.16; sandy soil: Wilks’ lambda = 0.70; *p* = 0.66; potting soil: Wilks’ lambda = 0.26; *p* = 0.12); however, differences between the treatments in terms of the water use efficiency of the plants were detected. In the cases of clay loam and peaty potting soil, which had high water holding capacities, the water retention effect of the wool mulch could be best measured in terms of the amount of total ET; this is because the water loss was 31.1% and 25.5% less, respectively, compared with the untreated control. However, this did not result in a larger amount of biomass growth on the sweet pepper seedlings in the short-term experiment. In the sandy soil, which had the lowest water capacity, the ability of the wool mulch to retain water was mainly exhibited by the fact that it produced a biomass that was 65% greater than that of the clay loam soil. 

The overall results showed that the wool mulch reduced physical evaporation in clay loam and potting soil, and it increased plant transpiration in sandy soil. The amount of water consumed per unit of biomass (ET_c_) was 38.2, 27.0, and 23.1% lower as compared with the control for the clay loam, sandy, and potting soils, respectively.

### 2.2. Wool Mulch–Plant Interaction Assessment in Greece

**Figure 1:**
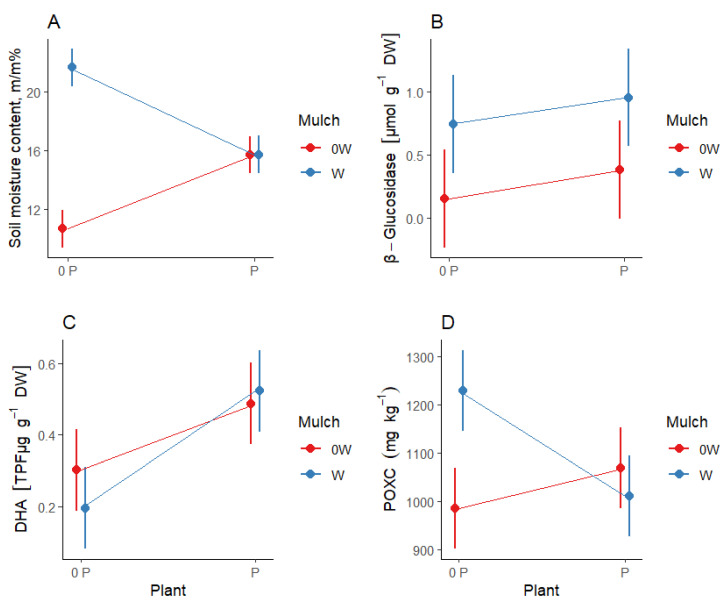
The effect of wool mulch and plant interactions at the Greek site. Interaction diagram showing the combined effect of two factors (mulch and plant; 0P: no plant. P: with plant; 0W: no mulch. W: with wool mulch) on the soil parameters. If there is no interaction, the lines run nearly in parallel. If there is an interaction, there is a difference in terms of the effect that the treatments have on the soil. Random fluctuations can affect the level of parallelism. If the figure seems to contradict the *p*-values that are associated with the interaction, prioritize taking the *p*-values into account.

During the Greek semi-field trial, which was characterized by a coarse loam soil, two factor levels (first factor: with plant, no plant; second factor: with wool mulch, no mulch plant) were tested in accordance with the four measured soil parameters. The effect of both single factors and their interactions were found to be significant (plant: Wilks’ lambda = 0.40; *p* < 0.01; mulch: Wilks’ lambda = 0.17; *p* < 0.001; interaction: Wilks’ lambda = 0.15; *p* < 0.001) (Table 2, Figure 1). 

Wool mulch had a positive significant effect on soil moisture retention and β-glucosidase activity. Regarding soil moisture, the partial eta squared value was high (η^2^ = 0.78), thus indicating that soil moisture was strongly influenced by the presence of mulch cover. The difference in β-glucosidase activity is significant, but the magnitude of the effect is much weaker (η^2^ = 0.30).

The most significant and highest level of soil moisture content was measured in the plots with wool mulch and without plants, whereas the lowest soil moisture content was found in the plots without mulch and plant. The effect of the plant on β-glucosidase activity was not detectable, whereas the activity of β-glucosidase was higher in the mulched plots, with or without the pepper plant, as compared with the non-mulched plots. The mulch had no significant effect on dehydrogenase enzyme activity (DHA); however, significantly higher DHA values were measured in the plots with plants. The effect of plant and mulch interactions with regard to permanganate-oxidizable soil carbon (POXC) was significant, and similar to the effect that the mulch had on soil moisture content. The plant–mulch interactions also have a high partial eta squared value (η^2^ = 0,78); this suggests that plant–mulch interactions have a strong and significant effect on soil moisture content. However, it must be noted that the strong partial eta squared value is due to the strong effect of the mulch on soil moisture content.

**Figure 2:**
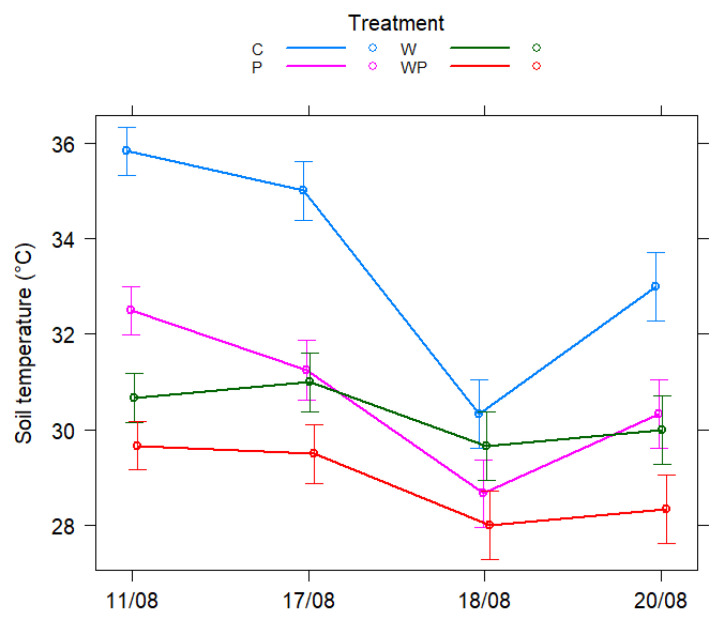
Soil temperature (°C) plotted as a function of treatments at different times at the site in Greece. In addition to the group averages (circles), 95% confidence intervals are given for each group based on the combination of treatment and time.

The positive effect of wool mulch on POXC was strongest when there were no plants in the plot. The soil temperature was also monitored during part of the growing season (Figure 2). The results clearly show that the soil temperature fluctuated more in the plots without wool mulch than in the plots that were covered with wool mulch. Soil temperature variance was particularly high in plots that were not mulched and which were without plants. At the Greek site, the yield of the mulched plots was, on average, 42% greater compared with the plots that were not mulched (Table 3). The sweet pepper yield was 1.3 kg per plant in plots that were subjected to the wool mulch treatment, whereas it was 0.9 kg in the case of the untreated control.

### 2.3. Comparison of Wool Mulch and Other Mulch Materials at the Serbian Site

**Figure 3:**
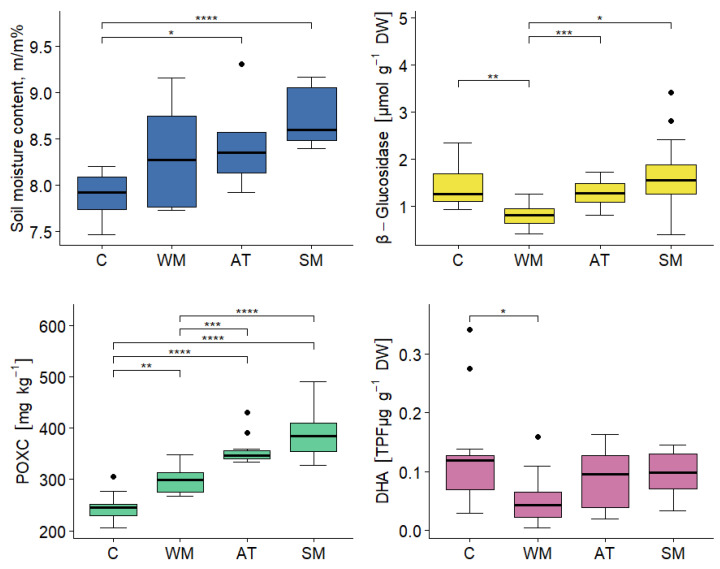
Summary of four measured soil variables at the Serbian site, with box and whisker plots indicating outliers and variations outside the lower and upper quartiles, as distinguished by the effect of the treatment: C: control; WM: wool mulch; AT: agro textile; SM: straw mulch. The results of the multiple comparisons of the UNIANOVA model are indicated by the lines above the graph. Statistical significance was determined using the Tukey’s (for soil moisture, POXC, and DHA) and Games–Howell (β-glucosidase) post-hoc tests, where ‘*’ *p* < 0.05 ‘**’ *p* < 0.01 ‘***’ *p* < 0.001 ‘****’ *p* < 0.0001. Post-hoc tests examine the homogeneity of variance.

The effect of three different mulch types on the four measured soil parameters was investigated in the Serbian semi-field trial. Treatments were significant for all four response variables (Wilks’ lambda = 0.10; *p* < 0.001, Figure 3). The different treatments significantly affected water retention capabilities; namely, the soil moisture content (F_3,44_ = 4.21; *p* < 0.001). In each mulch treatment, the observed soil moisture content was greater than the control (on average 7.89%, 8.32%, 8.44%, and 8.72% for the control, wool mulch, agro textile, and straw mulch treatments, respectively); however, the difference was only significant in the case of the straw mulch (*p* < 0.001). There were also significant differences between treatments in terms of β-glucosidase activity (F_3,44_ = 6.30; *p* < 0.01); however, none of the mulch treatments increased β-glucosidase activity to a greater level than the control. The wool mulch treatment exhibited significantly lower β-glucosidase activity than the control. Among the mulch treatments, straw mulch provided the greatest level of β-glucosidase activity on average (1.69 umol g^−1^ DW h^−1^); however, this is not significantly higher than the untreated control (mean 1.45 umol g^−1^ DW h^−1^). The treatments had the most significant effect on POXC among the four investigated parameters (F_3,44_ = 43.79; *p* < 0.001). The highest POXC level was observed in the case of the straw mulch treatment (mean 390.0 mg kg^−1^), followed by the agro textile and wool mulch treatments (means are 356.7 and 300.1 mg kg^−1^). A significant difference was also found in terms of DHA level (F_3,44_ = 3.23; *p* < 0.05). Recalling the effect of the treatment on β-glucosidase activity, the treatment had a similar effect on the DHA level, which was also significantly lower than the control when subjected to the wool mulch treatment (*p* < 0.05). Moreover, the other mulch treatments did not increase DHA compared with the control. 

At the Serbian site, the average yield per plant and the indicator values for soil microbiological activity were lower overall than at the Greek site for all treatments (Table 3). The greatest pepper yield was harvested after the plot was subjected to the straw mulch treatment (on average 0.85 kg per plant). The next greatest pepper yield was found in the agro textile plot, followed by the wool mulch plot, and finally, the control plot, with average weights of 0.81, 0.504, and 0.503 kg per plant, respectively.

## 3. Discussion

### 3.1. The Effect of Wool Mulch on Water Use Efficiency in Different Soil Types

The effect of wool mulch strongly depended on soil texture and the frequency of irrigation. In the pot experiment, the effect of the wool mulch on water use efficiency was also evident in the short-term, in all soil types. Wool mulching did not increase the sweet pepper yield or biomass in sandy soils with a low water capacity, nor did it increase the yield in the intensively irrigated open field plots of Serbia. It also failed to increase the yield in the extensively irrigated pot experiment. On the other hand, water use efficiency increased in the pot experiment, in the case of sandy soil; presumably, this was due to a decrease in physical evaporation and an increase in transpiration [34]. Regarding the Greek loam soil that had a good water capacity, as well as the clay loam soil and potting soil in the pot experiment, under extensive irrigation conditions, wool covering increased the efficiency of plant water use; this resulted in a greater yield at the Greek site, and a lower ET_c_ level in the pot experiment. In the sandy soil at the Serbian site, despite intensive irrigation, water may have been limited in limited supply even after the wool mulch treatment. Indeed, compared with the control, there was no increase in sweet pepper yield. This observation is consistent with the findings of previous research, according to which, the effect of mulching on biomass and yield becomes more significant as more time passes since irrigation, and when the water capacity of soils is greater [35,36]. Huang et al. [37] emphasized that the best water use efficiency can be achieved with the right combination of mulch type and watering frequency. In our case, the use of wool mulch can be effective, primarily with regard to soils that at least have a medium water capacity and when those soils are watered less frequently. In irrigated systems, the utilization of soil moisture is primarily determined by the water capacity of the soil, as well as the method and frequency of irrigation, and secondarily by the effect of mulching [16,36,37].

### 3.2. Effect of Wool Mulch–Plant Interactions on Soil Biological Activity

We found that the interaction between soil cover and the presence of plants is also important in the development of the moisture content of the soil and its biological parameters. At the Greek site, in the absence of pepper plants, the moisture retention effect of the wool mulch was greater and the soil moisture content remained high; however, in the presence of plants, due to transpiration, compared with the uncovered pepper plot, we did not measure a significantly higher soil moisture content that was due to the effect of the wool mulch cover. This difference in actual soil moisture content, as well as the interactions between the roots and the microorganisms, resulted in differences between the measured indicators of the biological activity of the soil. Indeed, despite a lower soil moisture content, the β-glucosidase activity was significantly higher in the presence of plants; this can be explained with the fact that it is not only water that plays a role in the development of the biological activity of the soil. Plant-based effects on the soil (i.e., metabolic products of roots) also play a major role [33,38]. This is confirmed by our result showing that the DHA level was higher in plots with pepper plants, likely because DHA also indicates root activity, in addition to the activity of microorganisms in the carbon cycle [39]. We did not observe a higher POXC level in the pepper plots that were covered with wool mulch compared with the uncovered pepper plots; this may indicate that the increased soil respiration rate may be due to the more intense microbiological activity, which reduced the easily oxidizable carbon sources by the end of the growing season [40]. The temporal trend exhibited by the biological parameters of the soil was also significantly influenced by the temperature of the soil [41]. In the pepper plots that were covered with wool mulch, the soil experienced the lowest temperatures and the smallest temperature fluctuations when compared with the other treatments. Together, the plants and the heat-insulating wool mulch provide a more favorable microclimate and less radiation reaches the soil. Moreover, a more balanced temperature allows for more balanced biological activity in the soil, which has also been confirmed by several other authors [1,16,17,29]. The role of the temperature of soil during the development of the biological activity of soil is also supported by the fact that Wang et al. [30] did not experience an increase in the biological activity of soil when black polyethylene (PE) mulch was used in their experiment; this has the highest water retention capacity out of the mulch materials, but it also has the effect of increasing the soil’s temperature. In Croatia, in a similar climate, Jungic et al. [15] found that covering plots with wool mulch resulted in a higher marketable lettuce yield than covering plots with black PE foil, even though the black PE had a better water retention capacity. Water is therefore a limiting factor for both plants and microbes; however, a more balanced soil temperature is also necessary to increase biological activity.

### 3.3. Effect of Wool Mulch on the Biological Activity of Soil in Comparison with Other Mulch Materials

Even with intensive irrigation and a nutrient supply, lack of water is a problem for sandy soil. At the Serbian site, the straw mulch had a significantly better water retention capacity compared with the agro textile mulch and the wool mulch; this was also reflected in the significantly higher soil moisture content and higher yield of sweet peppers. The positive effect of lignocellulose-based agricultural mulch materials on water use efficiency has already been reported by several authors [1,5,11,42]. A similar result was achieved by Duppong et al. [43], who found that the straw mulch treatment resulted in a higher crop yield than the wool mulch treatment. At the Serbian site, treatment with straw mulch, which decomposes more easily than wool mulch, resulted in the highest POXC level compared with the other mulch treatments; this finding is similar to the results found in Yu et al. [31]. Several authors have confirmed that in soils with weak biological activity, such mulch materials (e.g., straw mulch) are most effective in terms of water use efficiency and crop results; indeed, due to their faster decomposition rate, they contribute to the improvement of soils by increasing their organic matter content and nutrient retention capacity [10,13,14,18]. Although sheep wool is a valuable organic material and nutrient, it decomposes more slowly on the soil surface, and thus requires a hydrophilic environment for its decomposition [44]. In our experiment, although the agro textile was less effective in terms of retaining water, it did not significantly lag behind the straw mulch in terms of yield, presumably due to nitrogen immobilization during the decomposition of the straw mulch. Although wool mulch exhibited a soil water retention level that was better than the control, it produced less favorable results in terms of enzyme activity, and there was no difference in yield compared with the control. The reason for this could be due to the fact that continuous irrigation via sprinkler did not allow the wool mulch to dry and allow air to pass through; therefore, the resulting anaerobic conditions were unfavorable for both microbes and the pepper yield. As many authors have shown, the relationship between soil moisture content and enzyme activity (DHA, β-glucosidase) is not linear. Indeed, the optimal functioning conditions for soil microbes are as follows: a suitable air-water ratio, a good soil structure, and a sufficient amount of organic matter is required [28,32]. Moreover, in the case of the wool mulch treatment, the excess moisture did not result in a higher yield at the Serbian site due to the weaker biological activity in the soil. This is also consistent with the fact that the microbiological activity was generally higher at the Greek site than at the Serbian site. For example, the POXC level in the covered plot in Greece was double that of the level measured in the Serbian wool mulched plot, and the yield was also one and a half times higher in Greece; however, β-glucosidase activity was higher at the Serbian site, likely because the decomposition rate is higher in more oxidative sandy soils. Although, it must be noted that the amount of the most easily decomposable organic matter is likely to decrease faster [28,32].

## 4. Materials and Methods

### 4.1. Pot Trial

Each combination of soil type (sand, clay, potting soil) and wool mulch cover (yes/no) were set up and replicated 4 times, in a total of 24 samples. Non-woven wool mulch carpet, at a density of 500 g m^−2^ (Gartenwolle), comprising 100% washed sheep wool, was used for all the following experiments. The sandy soil came from the topsoil of Arenosols, which is typical of horticulture around Budapest (SOM = 1.60%; pH = 7.79). The clay soil came from the upper layer of a typical Gleysols in western Hungary (SOM = 2.53%; pH = 6.75). The potting soil was Pindstrup Plus, which is a typical peat-based medium for growing seedlings (pH = 5.9–6.0, NH_4_^+^ + NO_3_^−^ − N = 144 mg/L, P_2_O_5_ = 165 mg/L, K_2_O = 288 mg/L, EC = 1.2 dS/m). Air-dried soil samples were used to fill pots with a volume of 0.3 L and a diameter of 8 cm; they were allowed to absorb water through capillaries until the limit of their soil moisture capacity was reached. A gravimetric assessment was carried out to measure the soil water capacity and the total change in soil moisture. Sweet pepper ‘Amy F1’ (*Capsicum annuum* L.) seedlings were planted in each pot. The total dry biomass was also recorded at the end of the experiment. In addition, the soil moisture was measured using the digital soil moisture sensor, Huanyu type PMS710, on day 1, day 4, and day 10. Then, the soil was irrigated evenly once more, and measurements were continued on day 1, day 2, day 6, and day 10 when the experiment was completed. The total evapotranspiration (ET) was calculated in accordance with the change in total soil moisture content. The transpiration coefficients (ET_c_) were calculated as a ratio of total ET to dry biomass.

### 4.2. Semi-Field Trials

The semi-field experiments were carried out from June to September 2022 at two sites (a) to examine the effect that mulch cover (wool mulch only) has on extensive irrigation systems (Greece), soil parameters, and sweet pepper (‘Amy F1’) performance; and (b) to compare different mulching materials in an intensive system with no water restrictions (Serbia). Each treatment was performed and replicated four times (four plots per treatment), and the experimental design comprised a randomized block at both sites. In order to avoid soil heterogeneity, very small plots with a maximum area of 2 m^2^ were set up. The first site was in Greece, Sithonia (40.298524° N, 23.694660° E), which comprised a coarse–loamy neutral soil with a low–moderate organic matter content (Luvisols, SOM = 1.57%; pH = 6.90). A minimal amount of NPK (25 g m^−2^, NPK 14.5-7-14.5) fertilizer was added to the roots of the plants only. Weed growth was not relevant during the period of experimentation. Four plots measuring 1.32 m^2^ were set up with a combination of the following: wool mulch cover (yes/no) and the presence of a sweet pepper plant (yes/no). In cases where plants were added, 8 plants were placed in a grid, with a distance of 0.4 m between them. The plots were irrigated until their water capacity limit was reached, which became apparent when water remained on the surface of the soil. This occurred every 6 days leaving a dividing strip between the plots. The temperature of the soil was measured at a depth of 0–5 cm with a digital thermometer in the evening, so that the reading was taken without the direct radiation of the sun on the plots. The second site was in Serbia, Királyhalom (46.102046° N, 19.885812° E), which comprised sandy soil with a low soil organic matter content (Arenosols, SOM = 0.75%; pH = 7.44). Plots of 2 m^2^ (with no buffer area between them) were set up, comprising 10 sweet pepper plants per plot. Each plot was treated with intensive mineral fertilizers, and they were watered daily with 6 L m^−2^ of water using sprinklers. Weeds in the control plots were mechanically managed weekly. The effect of three mulching materials (wool mulch, agro textile, straw mulch) and a no-mulch control was assessed in accordance with the soil’s parameters and pepper yield. The agro textile mulch was a synthetic black PPHA foil that also allowed air, water, and fertilizer to sieve through the fabric for plant growth and to control weeds. The wool mulch was a non-woven wool mulch carpet with a density of 500 g m^−2^. The straw mulch material was composed of wheat straw. Before planting, farmyard manure was added to the entire surface of the plots at a dose of 6 kg m^−2^.

Based on data from the Meteoblue database, the summer period (June to September) in both locations tends to be hot and dry (Table 4). In Greece, the average daily temperature per month was higher (25.5 °C, 27.24 °C, 27.99 °C, and 22.32 °C, respectively) than in Serbia (25.34 °C, 26.36 °C, 26.33 °C, and 18.45 °C, respectively). There was not a great deal of rainfall in the summer (5.5 mm, 3.9 mm, 39.7 mm, and 51.1 mm, respectively) and the relative humidity was also low at the Serbian site (41.66% 35.7%, 43.79%, and 57.98%, respectively). Overall, this resulted in lower predicted evapotranspiration values (1.78 mm, 0.17 mm, 0.49 mm, and 1.00 mm, respectively) than at the Greek site (2.28 mm, 1.51 mm, 1.05 mm, and 0.95 mm, respectively). At the Greek site, the relative humidity was relatively high (55.27%, 45.72%, 49.08%, and 60.35%, respectively) and the total precipitation level per month was also low (22 mm, 39.8 mm, 13.4 mm, and 51.1 mm, respectively).

Composite soil samples per plot, at a depth of 0–10 cm were taken 3 times during the semi-field experiments to monitor soil biological activity. The β-glucosidase and dehydrogenase (DHA) enzyme activity, as well as the level of permanganate oxidizable active carbon (POXC), was measured to assess the biological activity of the soil in two of the plots for each sample. To measure the β-glucosidase activity, we used the method given by Sinsabaugh et al. [45] with a minor modification, in accordance with the investigation by Kotroczó et al. [39]; this modification involved increasing the concentrations of the buffer and terminal solutions. Dehydrogenase activity (DHA) was measured with the reduction of 2.3.5-triphenyltetrazolium chloride method, as proposed by Veres et al. [46]. The POXC level was measured in accordance with the method given in Weil et al. [47], wherein the change in concentration level of KMnO_4_ was used to estimate the amount of carbon that was oxidized. This method was modified by shaking of 1 g of air-dried soil in 10 mL 0.02 M KMnO_4_ solution for 5 min, and the absorbance level was measured at a wavelength of 565 nm using a Biochrom Libra S22 spectrophotometer (Biochrom Ltd., Cambridge, United Kingdom). The soil moisture content of the soil samples was measured gravimetrically. The crop yield harvested from the plots was recorded.

### 4.3. Data Analysis

All statistical analyses were performed within the R (v. 4.2.1) statistical environment. The multivariate analysis of variance was used to investigate the effect of treatments for the response variables. The treatments in the pot experiment were compared according to soil type. For all three response variables, normality was accepted in accordance with the Kolmogorov–Smirnov test. The variance homogeneities for the response variables were tested using Levene’s test, and they were only corrupted to assess the evapotranspiration and ET_c_ levels; however, the ratios of maximum and minimum variances were accepted [48]. At the Greek site, the plant–mulch interaction was tested using the two-way MANOVA model for the four response variables. The effect of the two factors was also assumed to be Wilks’ Lambda for the target variables. In cases where a significant effect was detected, the univariate main effects were also tested in conjunction with a Bonferroni correction. We evaluated the partial η^2^ value, which gives the effect size as well as the observed power; moreover, this indicates the likelihood of detecting the correct significant effect. The value of the indicator can range from 0 to 1. Zero indicates independence and 1.00 indicates a deterministic relationship. The interaction between the two factors was also examined in the model. To visualize the interaction, we used the effects package [49]. For all four response variables, normality was assumed on the basis of the Shapiro–Wilk test. The homogeneity of variances was also checked using Levene’s test. The effect of the treatments on the measured soil parameters at the Serbian site was evaluated using one-way MANOVA, and the effectiveness of the treatments was assessed in accordance with Wilks’ Lambda. Given the significance of the overall test, the univariate main effects were examined in conjunction with Bonferroni’s correction. The assumption of the normality of the residuals was checked using the Shapiro–Wilk test and the kurtosis and skewness test [50]. The homogeneity of variances was checked using Levene’s test.

## 5. Conclusions

We investigated the effect of mulching in two areas with similar arid climates and temperatures that are suitable for pepper cultivation. The different water holding capacities of the soils was of great importance to the results, and we found that the water holding capacity also affects the required irrigation intensity. In the case of soils with at least a medium water holding capacity (Greek site), wool mulch is a sustainable practice for improving the physical and soil biological parameters of soil and enabling water management to become more efficient. Regarding soils with low water capacities and low organic matter contents (Serbian site), frequent watering is inevitable; therefore, wool mulch is more suitable for fields with soils that have a baseline medium water capacity, and for extensive irrigation systems that allow aeration and microbiological activity to occur in the root of the plant. In the case of intensively irrigated sandy soils, using organic mulch materials (e.g., straw) that decompose faster are better suited to such conditions than wool mulch. The effect of WM on crop yields was explained by the water retention of the mulch and the development of the soil’s biological activity, as well as plant–soil–microbe interactions. Our results show that β-glucosidase and POXC levels are excellent indicators of the effect of mulching and the differences in crop yields. In addition to the physical parameters of the soil, monitoring the soil–plant–microbe relationship is also very important for selecting the most suitable mulch material for the given environmental conditions.

## Figures and Tables

**Table 1:** The effect of wool mulch on total evapotranspiration (ET), biomass growth, and evapotranspiration coefficients (ET_c_) in different soil types in a short-term pot experiment (mean ± standard deviation).

Soil Type	Treatment	Total ET, g		Dry Biomass per Plant, g		ET_c_, g Water g^−1^ Dry Biomass	
Clay loam	C	89.65	±19.12	0.30	±0.11	331.47	±137.91
	WM	61.80	±20.96	0.32	±0.06	204.82	±84.17
Sandy soil	C	80.58	±25.03	0.18	±0.038	457.84	±131.28
	WM	81.77	±26.54	0.30	±0.19	334.10	±156.85
Potting soil	C	126.35	±13.12	0.64	±0.08	198.14	±10.71
	WM	94.12	±20.78	0.61	±0.06	152.28	±21.31

C: untreated control; WM: wool mulch treatment.

**Table 2:** The effect of the wool mulch and plant interactions at the Greek site. Results showing the tests of between-subject effects (UNIANOVA).

Predictors	Response Variable	F-Value	Sig Level	Effect Size (η^2^)
Mulch	Soil moisture	72.11	***	0.78
	β-Glucosidase	8.68	*	0.30
	DHA	0.37	ns	0.02
	POXC	4.80	ns	0.19
Plant	Soil moisture	0.47	ns	0.02
	β-Glucosidase	1.24	ns	0.06
	DHA	19.29	***	0.49
	POXC	2.51	ns	0.11
Mulch ∗ Plant	Soil moisture	71.58	***	0.78
	β-Glucosidase	0.002	ns	0.00
	DHA	1.46	ns	0.07
	POXC	12.57	*	0.39

‘*’ *p* < 0.05 ‘***’ *p* < 0.001. Given the significance of the overall test, the univariate main effects were investigated using the Bonferroni correction method.

**Table 3:** The effect of treatments on average pepper yield per plant at the Serbian and Greek sites.

Site	Treatment	Average Sweet Pepper Yield (kg per Plant)
Serbian site (sandy soil)	Control (C)	0.503
	Straw mulch (SM)	0.850
	Agro textile (AT)	0.808
	Wool mulch (WM)	0.504
Greek site (coarse loam)	Plant with wool mulch (PW)	1.283
	Plant without wool mulch (P)	0.904

**Table 4:** Summary of meteorological data at the semi-filed trial locations (2022).

Location	Date	Average Daily Temperature (°C)	Total Amount of Precipitation (mm)	Humidity (%)
Greek site Coarse–loamy neutral soil with low–moderate organic matter content: SOM = 1.57%; pH = 6.90	June	25.5 °C	2.28 mm	55.27%,
	July	27.24 °C	1.51 mm	45.72%,
	August	27.99 °C	1.05 mm	49.08%,
	September	22.32 °C	0.95 mm	60.35%
Serbian site Sandy soil with low soil organic matter content: SOM = 0.75%; pH = 7.44	June	25.34 °C	5.5 mm	41.66%
	July	26.36 °C	3.9 mm	35.7%
	August	26.33 °C	39.7 mm	43.79%
	September	18.45 °C	51.1 mm	57.98%

## Data Availability

The data presented in this study are available upon request from the corresponding authors.
